# All-atoms MD simulations study of newly designed ciprofloxacin derivatives as potential bacterial DHPS and DNA gyrase inhibitors

**DOI:** 10.3389/fbinf.2026.1876643

**Published:** 2026-09-03

**Authors:** Chita Ranjan Sahoo, Madhusmita Rout, Sudhir Kumar Paidesetty, Budheswar Dehury, Debdutta Bhattacharya, Sanghamitra Pati

**Affiliations:** 1 Department of Health Research, ICMR-Regional Medical Research Centre, Ministry of Health and Family Welfare, Government of India, Bhubaneswar, India; 2 Medicinal Chemistry Research Laboratory, School of Pharmaceutical Sciences, Siksha ‘O’ Anusandhan Deemed to be University, Bhubaneswar, India; 3 Department of Bioinformatics, Manipal School of Life Sciences, Manipal Academy of Higher Education, Manipal, Karnataka, India; 4 Indian Council of Medical Research, New Delhi, India

**Keywords:** AMR (antimicrobial resistance), ciprofloxacin, molecular docking, molecular simulation, sulfanilamide

## Abstract

The widespread use of antibiotics, particularly ciprofloxacin, has accelerated the emergence of antibiotic-resistant bacteria, posing a major public health challenge. Limited investment and declining pharmaceutical interest have slowed the discovery of new antibiotics, contributing to the increasing prevalence of multidrug-resistant (MDR) infections, especially those caused by ESKAPE pathogens. Uropathogenic *Escherichia coli* (UPEC), a leading cause of urinary tract infections (UTIs), develops multidrug resistance through mechanisms including drug target modification and reduced antibiotic susceptibility. In the present study, a series of ciprofloxacin-sulfonamide hybrid molecules (CIS1-CIS15) was designed, in which each pharmacophore retains its established mechanism of action. Ciprofloxacin inhibits DNA gyrase and topoisomerase IV, whereas sulfonamides inhibit dihydropteroate synthase (DHPS) by competing with para-aminobenzoic acid (PABA) in the bacterial folate biosynthetic pathway. Accordingly, the designed hybrids were computationally evaluated against both enzymatic targets to investigate their potential dual-target antibacterial activity. Molecular docking and molecular dynamics (MD) simulations were performed to assess the binding affinity and stability of the ciprofloxacin-bearing sulfonamide derivatives (CIS1-CIS15) against DNA gyrase and *Staphylococcus aureus* DHPS. Principal component analysis (PCA) and free-energy landscape (FEL) analysis were used to characterize the conformational flexibility and low-energy states of the protein–ligand complexes, while MD simulations validated the stability of the highest-scoring docked complexes. Among the designed derivatives, CIS4 (ciprofloxacin-sulfamethoxazole), CIS5 (ciprofloxacin–sulfaguanidine), and CIS12 (ciprofloxacin-sulfamerazine) exhibited the strongest binding affinity toward both targets while maintaining conformational stability throughout the simulations. Structural analysis of the pre- and post-MD trajectories identified Arg52, Lys39, Arg88, Lys94, Lys120, Glu201, and Lys203 as key residues involved in ligand recognition. Overall, CIS4, CIS5, and CIS12 emerged as the most promising dual-target inhibitor of bacterial DHPS and DNA gyrase. These findings provide valuable structural insights for the rational design of novel fluoroquinolone-based antibacterial agents and demonstrate the utility of computational approaches in identifying potential therapeutics against drug-resistant bacterial pathogens.

## 1 Introduction

*Staphylococcus aureus* (*Staphylococcus aureus*) is a Gram-positive bacterium implicated in numerous infections in both community and hospital settings. It is among the most prevalent opportunistic human pathogens and is associated with high morbidity and mortality rates (Abbasian et al., 2018). The rate of *S. aureus* colonization is estimated to be approximately 30%, occurring predominantly on the skin and nasal mucosa (Monaco et al., 2016). This pathogen can infect virtually all human tissues, leading to a wide range of diseases, including bacteremia, infective endocarditis, skin and soft tissue infections, osteomyelitis, arthritis, pneumonia, empyema, meningitis, and urinary tract infections. *S. aureus* expresses various virulence factors, such as surface proteins that facilitate bacterial adhesion, extracellular enzymes, and toxins that induce tissue necrosis, along with immune evasion mechanisms (Lowy, 1998). The bacterium produces several enzymes, including coagulase, hyaluronidase, deoxyribonuclease, and lipase, which contribute to its pathogenicity and dissemination within the host (Tam and Torres, 2019). As a member of the ESKAPE group of pathogens, which includes *Enterococcus faecium*, *Klebsiella pneumoniae*, *Acinetobacter baumannii*, *Pseudomonas aeruginosa*, and *Enterobacter species*, *S. aureus* is among the most challenging pathogens to treat because of its multidrug resistance (Bereanu et al., 2024; Boucher et al., 2009). Staphylococci, particularly *S. aureus*, are the leading cause of device-related infections and are associated with more severe and aggressive clinical manifestations (Darouiche, 2001).

**SCHEME 1:**
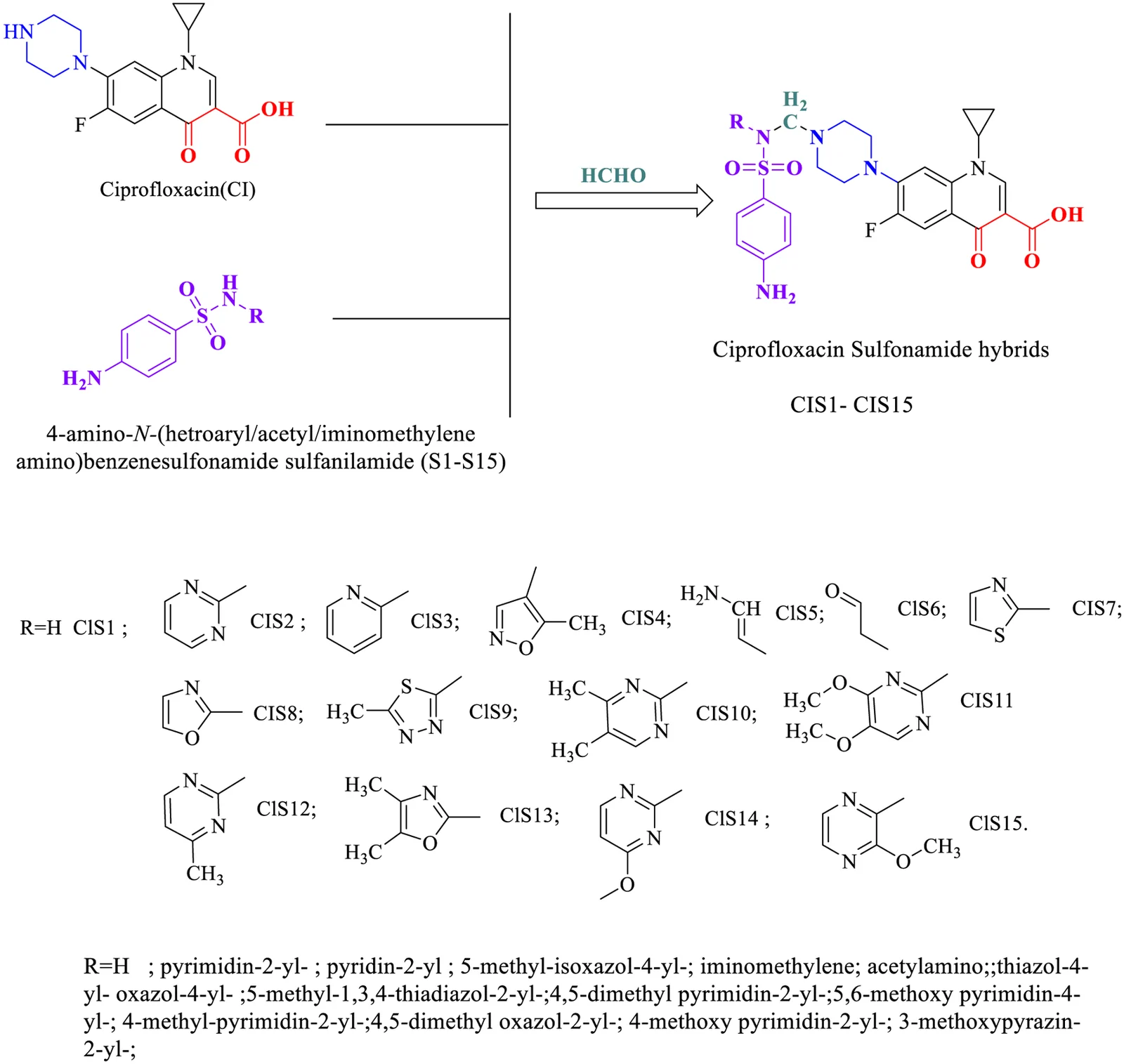
Schematic approaches for newly designed ciprofloxacin-sulfonamide hybrids.

The 2022 World Health Organization (WHO) report highlights the global prevalence of AMR, which poses a significant threat to individuals of all ages and geographic regions. The consequences of unchecked AMR are far-reaching and affect not only global health but also food security, environmental wellbeing, and socioeconomic development. The key cellular mechanisms responsible for AMR include enzymatic hydrolysis, antibiotic modification via group transfer and redox processes, alteration of antibiotic targets, reduced antibiotic permeability through porin modification, and active efflux via membrane pumps (Cox and Wright, 2013; Fajardo et al., 2008).

*S. aureus* has rapidly developed resistance to multiple antibiotics, presenting a major challenge in treating infections (Kherroubi et al., 2024). The bacterium produces antibiotic-degrading enzymes, contributing to a variety of resistance mechanisms (Egorov et al., 2018). These enzymes play pivotal roles in bacterial survival under antibiotic pressure, promoting the genetic diversity, evolution, and spread of resistant strains. The widespread use of antibiotics in human medicine, agriculture, and the environment has created strong selection pressures, facilitating the emergence and proliferation of resistant clones (Larsson and Flach, 2022). Resistance to multiple antibiotics renders *S. aureus* a particularly difficult pathogen to manage. Methicillin-resistant *S. aureus* (MRSA) and vancomycin-resistant *S. aureus* (VRSA) are among the most clinically significant resistant strains. MRSA, in particular, is recognized as a high-priority pathogen because of its potential to cause substantial global mortality in the absence of effective treatment strategies (Khan et al., 2018; Purrello et al., 2016). The increasing prevalence of multidrug-resistant strains, including VRSA, is a persistent public health concern, with antibiotic-resistant infections now causing more than 10 million deaths annually, a figure expected to surpass cancer-related mortality by 2050 (Walsh, 2000).

Among all classes of antibiotics, fluoroquinolones are among the most widely used because of their high efficacy against uropathogenic bacteria, which they target by inhibiting bacterial DNA gyrase. Fluoroquinolones exert their antibacterial effects by inhibiting DNA gyrase and topoisomerase IV activity, stabilizing the enzyme-DNA cleavage complex and causing double-stranded DNA breaks that lead to bacterial cell death; resistance mechanisms involve mutations in gyrA and parC, efflux pumps, and Qnr proteins, as supported by recent findings (Coba-Males et al., 2023; Kherroubi et al., 2024).

Currently, research is focused on developing novel fluoroquinolone derivatives to combat resistance and enhance the antibacterial spectrum. Several studies in the literature have reported that structural alterations in fluoroquinolones, viz. Ciprofloxacin, norfloxacin, and levofloxacin, especially at the C-7 position of the piperazinyl side chain, have shown enhanced activity against uropathogenic bacteria *via* Mannich reactions or the introduction of heterocyclic rings and chalcone derivatives (Ruiz-Marcial et al., 2010).

SAR studies indicate that the presence of a keto group and a free carboxylic group at the C-3 and C-4 positions, respectively, is crucial for effective binding to DNA gyrase and topoisomerase IV. This interaction disrupts bacterial DNA replication, leading to the increased incorporation of false nucleotides and ultimately halting protein synthesis, which results in the death of the bacterial cell. Among the older classes of antibacterial agents, sulfonamides are structural analogs of para-aminobenzoic acid (PABA), and they have become largely obsolete because of the widespread development of bacterial resistance (Ghomashi et al., 2023). However, these are still used in combination with other antibacterial agents to achieve a synergistic effect. Sulfonamides act by inhibiting bacterial folic acid biosynthesis, an essential pathway for nucleotide and DNA synthesis. The development of novel fluoroquinolone-class antimicrobial drugs combined with sulfonamide moieties may offer promising approaches for addressing the challenge of antimicrobial resistance (Fedorowicz et al., 2019, Fedorowicz et al., 2023; Fedorowicz and Sączewski, 2018), which contributes to prolonged illness, increased healthcare costs, and increased mortality rates. Resistance to nearly all commonly used antibiotics in clinical practice has been reported, but only a limited number of new drugs are currently in development (Ghomashi et al., 2023). This highlights the urgent need for research focused on both the discovery of novel antibiotics and the enhancement of infection control protocols. The primary objective of this endeavour is to identify and develop potent lead quinolone candidates with improved efficacy against resistant pathogens. In light of these resistance challenges, we propose the development of hybrid antibiotic compounds that combine ciprofloxacin derivatives and sulfanilamides to form a single molecular entity.

Mannich-based ciprofloxacin-bearing sulfanilamides are designed with various substituents on their molecular structure to optimize the inhibition of DNA gyrase and *S. aureus* dihydropteroate synthase, two essential bacterial enzymes involved in DNA replication and folate synthesis, respectively. The selection of these substituents, often aromatic or heterocyclic groups with electron-withdrawing or electron-donating properties, is guided by structure‒activity relationship studies, which demonstrate that such modifications increase binding affinity and antimicrobial efficacy, particularly against resistant strains. Our selection rationale is rooted in both experimental results and molecular simulations, which demonstrate that compared with ciprofloxacin alone, these specific designs result in enzyme inhibition and reduced toxicity, supporting the development of effective action against bacteria (Ibrahim et al., 2022; Sahoo et al., 2025). This strategy aims to overcome bacterial resistance by leveraging the mechanisms of action of both drug classes. The hybridization of designed ciprofloxacin-sulfanilamide molecules is based on SAR studies and the mechanisms of action of ciprofloxacin derivatives and sulfanilamides. These hybrid molecules are designed to exhibit dual inhibitory activity, targeting both bacterial DNA gyrase and dihydropteroate synthase (DHPS), thereby mimicking the antibacterial effects of sulfonamide drugs that act against specific DNA gyrase enzymes. We selected DHPS and DNA gyrase as the primary targets for *in silico* studies to assess the potential of these hybrid compounds in overcoming resistance.

This study aims to investigate the kinetics and conformational states of the DNA gyrase enzyme when it is bound to novel ciprofloxacin derivatives via advanced computational techniques. Our structural function analysis serves as a benchmark for the development of new, broad-spectrum gyrase inhibitors. The ongoing challenge of developing new antimicrobial agents is underscored by widespread resistance to existing antibiotics, which results in longer illness durations, higher healthcare costs, and increased mortality rates. With limited new drugs in the pipeline, this research contributes to the discovery of novel antibiotics and the enhancement of infection control strategies, which are crucial for addressing the growing threat of antibiotic-resistant pathogens.

## 2 Materials and methods

### 2.1 Preparation of the receptor and library

Dihydropteroate synthase (apo form) from *S. aureus* (1AD1), topoisomerase IV subunit A (GrlA) from *Staphylococcus aureus* (2INR), and *S. aureus* gyrase B cocomplex with methyl ({5-[4-(4-hydroxypiperidin-1-yl)-2-phenyl-1,3-thiazol-5-yl]-1 h-pyrazol-3-yl} methyl) carbamate inhibitors (3G7B) constitute the X-ray crystallographic structures of the main drug targets of UTI bacteria. The Protein Preparation Wizard (Madhavi Sastry et al., 2013) of the Epik module of the Schrödinger suite (Schrödinger, LLC, New York, 2021–22) was then used to prepare the proteins. After the protein structure was preprocessed and the model was optimized and minimized, hydrogen bonds were included, along with hydrogen bond optimization with the PROPKA module (Shelley et al., 2007).

In the Mannich reaction, the fluoroquinolone derivatives CIS1-CIS15 (Scheme 1) were joined with sulfanilamide drugs, viz. Sulfanilamide, sulfadiazine, sulfapyridine, sulfamethoxazole, sulfaguanidine, sulfacetamide, etc., are present at the C-7 position of the piperazinyl side chain via a methylene linker without altering keto or carboxylic groups (REF b). These hybrids bearing Oxo and free carboxylic groups freely interact with or bind to DNA gyrase and compete with their PABA analogs (Ghomashi et al., 2023). ChemBioDraw Ultra and ACD-Labs ChemSketch were used to construct and optimize the 2D structures of the ciprofloxacin derivatives (CIS1-CIS15), and Open Babel was used to minimize energy (Supplementary Table S1). The pharmacokinetic characteristics of the proposed compounds were subsequently assessed. The LigPrep tool in Maestro 12.8 was used to prepare ligands for molecular interaction research. The OPLS4 force field (Lu et al., 2021) was used to create energy-minimized structures at pH 7.0 ± 2.0.

### 2.2 Molecular docking

For molecular docking, an active-site residue-based binding pocket selection approach was employed using the experimentally validated ligand-binding residues reported for each target protein rather than defining a grid box by explicit center coordinates and dimensions. The Glide docking program from the Schrodinger suite (Schrodinger, LLC, New York) was used to examine the molecular interactions between ciprofloxacin derivatives and the targets DHPS and DNA gyrase. Standard precision (SP) mode was used for molecular docking calculations first, followed by extreme precision (XP) mode. The docked complexes of DHPS, DNA gyrase, and ciprofloxacin derivatives were displayed via PyMOL and BIOVIA Discovery Studio v2021. In Prime (Schrodinger, LLC, New York), the molecular mechanics-generalized born surface area (MM/GBSA) approach was used to determine the binding free energy (ΔGbind) of the ligands at the ligand binding site of both targets (Rout et al., 2024). The researchers provided a detailed explanation of the approach used to calculate the MM/GBSA (Genheden and Ryde, 2015; Jawarkar et al., 2022).

$$\Delta G_{\text{bind}}=G_{\text{complex}}-G_{\text{receptor}}-G_{\text{ligand}}$$

$$\Delta G_{\text{bind}}=\Delta H-T\Delta S\approx \Delta E_{\text{gas}}+\Delta G_{\text{sol}}-T\Delta S$$

The best binding interactions of the complexes with the highest scores in the SP, XP, and ΔG_bind_ tests were chosen for additional screening (Sahoo et al., 2020). However, recently, the protein-peptide docking with a developing generative model for cellular signalling for therapeutic development has several methodological limitations (Zhao et al., 2025).

### 2.3 Molecular dynamic simulation and trajectory analysis

Using the CHARMM36m (Huang and MacKerell, 2013) force field and the TIP3P (Jorgensen et al., 1983) water model, we ran all-atom MD simulations in GROMACS to assess the flexibility and stability of the docked complexes. To create ligand topologies, the CHARMM general force field (CGenFF) was employed (Vanommeslaeghe and MacKerell, 2012). After each system was neutralized with 0.15 M NaCl, the steepest descent technique was used to minimize energy over 5000 steps. For the NVT and NPT ensembles with periodic boundary conditions, the minimized systems were equilibrated via Berendsen thermostats and Nose‒Hoover (Hoover, 1985; Nosé, 1984). At 303 K and 1 bar of continuous pressure, a 200 ns production MD simulation was performed with a Parrinello-Rahman barostat (Parrinello and Rahman, 1981).

Trajectory snapshots, generated every 0.2 ps during the simulation period, were analyzed in detail via the GROMACS analysis toolkit. The tools *gmx rms*, *gmx rmsf*, *gmx gyrate*, *gmx sasa*, and *gmx hbond* were used to calculate key properties, including the backbone RMSD, Cα RMSF, radius of gyration (Rg), solvent-accessible surface area (SASA), and intermolecular hydrogen bonding. In 200 ns MD simulations, VMD was used to map time-dependent structural changes in secondary elements. Plots were created with Adobe Illustrator, while 2D graphs and structures were generated using XmGrace, PyMOL, and Discovery Studio Visualizer.

Principal component analysis (PCA) was carried out in conjunction with *gmx covar* and *gmx anaeig* to separate collective motions from local dynamics in the complexes. RMSD-based clustering was performed using a gmx cluster with a 0.2 nm cutoff for the final 100 ns of the MD trajectory.

## 3 Results and discussion

### 3.1 Molecular docking studies

To identify potential inhibitors of both DHPS and DNA gyrase, molecular docking was performed using Glide (Panda et al., 2025) implemented in Schrodinger. Screening of fifteen newly developed compounds (Scheme 1). These compounds were then rescreened and scored based on their Glide XP and MM/GBSA values. Table 1 presents the binding affinity and MM/GBSA scores for the top-ranked postures, along with the residues involved in nonbonded interactions.

**TABLE 1:** List of ciprofloxacin derivatives that have relatively high binding affinities and MM/GBSA binding free energies with DHPS and DNA gyrase. The critical amino acids that form crucial nonbonded contacts with ligands are also listed in bold.

Receptor	Ligands	SP score (kcal/mol)	XP score (kcal/mol)	MMGBSA (kcal/mol)	H-bond	Hydrophobic	Electrostatic
**1AD1**	CIS1	−3.40	−4.21	−39.80	**Arg52**, **Arg239**, **Asn11**, Ser16	His241	Arg52, Lys203, Arg239, Arg32
	CIS2	−3.87	−3.98	−41.61	**Arg52**, **Arg239**, Arg219, Arg202	Lys203	Arg219
	CIS11	−3.09	−4.22	−41.07	**Arg52**, Ser18, Gln105, Arg202, **Arg239**, **Asn11**	Asn11, Phe17, His241, Val217	Arg32, Arg52, Arg239, Lys203
	CIS5	−2.97	−3.65	−54.14	Gln105, Arg219, Lys207, Arg239	His55, Arg239	Arg239, Lys207
	CIS12	−3.13	−5.36	−41.38	Ser18, **Arg52**, **Arg239**, **Asn11**, Ser16	Phe17, His241, Pro216	Arg52, Arg239, Lys203
**2INR**	CIS5	−3.42	−5.44	−35.10	**Ala30**, Gln91, **Lys94**, Pro114, Glu84	Glu84, Arg88, Pro40	**Lys94**
	CIS12	−4.04	−5.38	−28.88	**Ala30**, Gln91, **Lys94**	Glu84, Arg88, Pro40, Val41	**Lys94**, Glu84, **Ala30**
**3G7B**	CIS1	−3.53	−4.36	−42.01	Asn54, Arg144, Glu50, Arg84, Ser129	**Ile86**, **Pro87**	Arg84, **Glu58**, Arg144
	CIS4	−4.65	−5.10	−35.24	Val130, Thr173, **Asp81**, Asn54	Asn54, Ile102, **Ile86**	Glu50
	CIS5	−5.07	−5.42	−35.37	Gln91, Asn54, **Asp81**	**Pro87**, Ala98, Ile86	**Glu58**

**FIGURE 1:**
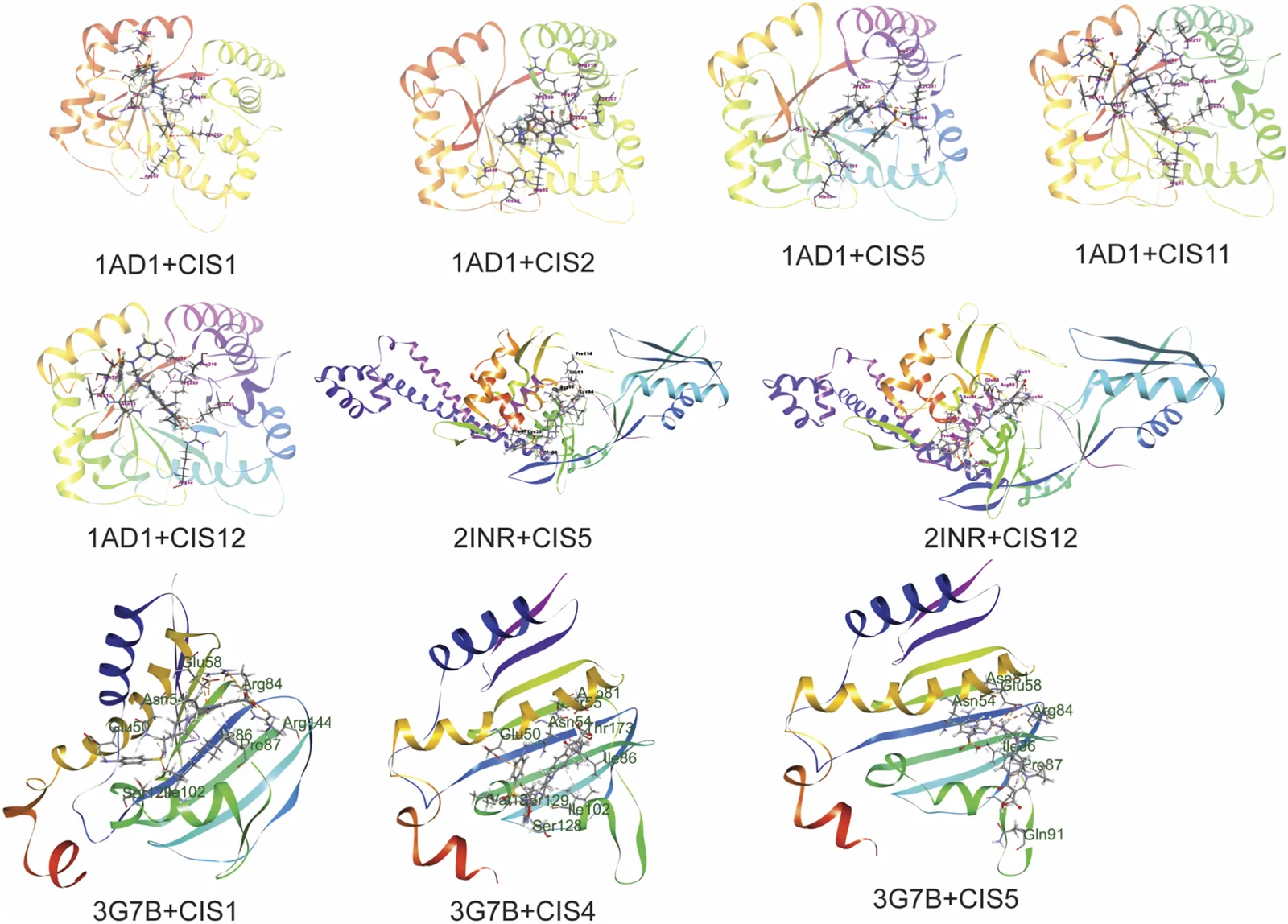
Nonbonding interactions between ciprofloxacin–sulfonamide hybrid derivatives and DHPS and DNA gyrase were determined by analyzing the top-ranked complexes obtained from molecular docking studies. The protein is depicted in cartoon representation, while the ligands are shown as stick models, with interacting amino acid residues labeled. Dashed lines indicate different types of noncovalent interactions. Binding interactions are shown for dihydropteroate synthase (DHPS, 1AD1), DNA gyrase (2INR), and DNA gyrase (3G7B) with the ciprofloxacin–sulfonamide hybrid derivatives.

For 1AD1, the XP scores for CIS1, CIS2, CIS11, CIS5, and CIS12 were −4.21, −3.98, −4.22, −3.65, and −5.36 kcal/mol, respectively, whereas the binding energies were −39.80, −41.61, −41.07, −54.14, and −41.38 kcal/mol, respectively. CIS5 and CIS12 had binding energies of −5.44 and −5.38 kcal/mol, respectively, and −35.10 and −28.88 kcal/mol, respectively. In 3G7B, the binding energies of CIS1, CIS4, and CIS5 were −4.36, −5.10, and −6.42 kcal/mol, respectively, with corresponding binding scores of −42.01, −35.24, and −35.37 kcal/mol, respectively. In 1AD1, key contacts included hydrogen and electrostatic connections formed by Arg52 and Arg239, as well as hydrophobic contacts made by His241. In the 2INR, Glu84, Arg88, and Pro40 established hydrophobic contacts, whereas Ala30, Gln91, and Lys94 formed hydrogen-bonding and electrostatic interactions. In 3G7B, Ile86 and Pro87 formed hydrophobic contacts, whereas Asn54 formed hydrogen bonds (Figure 1).

**FIGURE 2:**
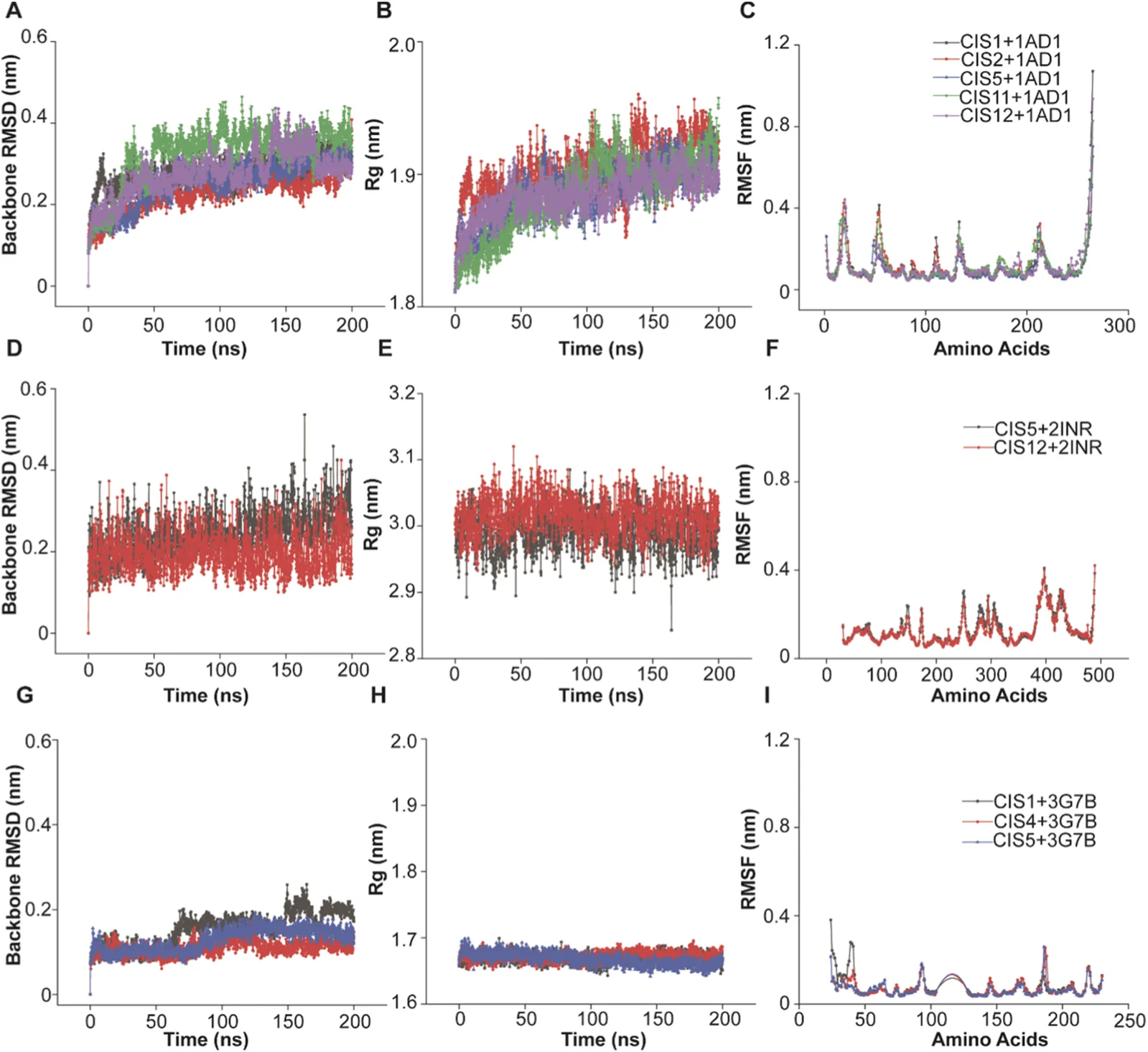
The dynamic stabilities of the ciprofloxacin-sulfonamide hybrid derivatives with DNA gyrase activity and dihydropteroate synthase activity were assessed via all-atom MD. **(A)** Backbone RMSD of dihydropteroate synthase (PDB: 1AD1) and ciprofloxacin-sulfonamide hybrid derivative (CIS1, CIS2, CIS5, CIS11 and CIS12) complexes. **(B)** DHPS complex profile of radius gyration. **(C)** C-alpha RMS fluctuation plot of dihydropteroate synthase and ciprofloxacin-sulfonamide hybrid derivative complexes. **(D)** Backbone RMSD of DNA gyrase (PDB:2INR)-ciprofloxacin-sulfonamide hybrid derivative (CIS5 and CIS12) complexes over a time period of 200 ns. **(E)** DNA gyrase complex profile of radius gyration. **(F)** C-alpha RMS fluctuation plot of DNA gyrase and ciprofloxacin-sulfonamide hybrid derivative complexes. **(G)** Backbone RMSD of DNA gyrase (PDB:3G7B)-ciprofloxacin-sulfonamide hybrid derivative (CIS1, CIS4 and CIS5) complexes over a time scale of 200 ns. **(H)** DNA gyrase complex profile of radius gyration. **(I)** C-alpha RMS fluctuation plot of DNA gyrase and ciprofloxacin-sulfonamide hybrid derivative complexes (CIS1, CIS4 and CIS5).

Supplementary Figure S1 is a 2D, while Figure 2 is a 3D illustration of the nonbonded interactions between these protein–ligand complexes. The role of hydrogen bonding in molecular recognition and complex formation has been demonstrated in earlier studies (Dey et al., 2024; Panda et al., 2025; Rout et al., 2024; Sahoo et al., 2025). Additionally, the study of the intermolecular interactions of the docked complexes suggests a role in the molecular recognition process. However, moxifloxacin was recently reported to target *S. aureus* DNA gyrase (PDBID: 5CDQ), which binds to ±1 bases of the DNA cleavage site of the protein and blocks the scissile phosphate bond to the 3′-hydroxyl of the cleaved DNA, thereby inhibiting DNA religation (Chan et al., 2015). Similarly, *K. pneumoniae* quinolone-stabilized cleavage complex of topoisomerase IV (PDBID: 5EIX) is a targeted antibiotic against levofloxacin, where the active binding sites are Lys442, Gln460, Glu461, Asp421, Ser80, Glu84, Glu419, Asp491, Asp493, Tyr120, and Arg119 (Veselkov et al., 2016). Our study revealed that the top-ranked ciprofloxacin derivatives strongly bind to the active site of the bacterial protein (5CDQ and 5EIX), which is recognized as having important interactions with crucial residues, such as *S. aureus* DNA gyrase with Ser84 and *K. pneumoniae* with Asp464, Gln460, Glu461, Lys442 and Gly443.

### 3.2 Dynamics of ciprofloxacin-sulfonamide hybrid derivatives complexed with DHPS and DNA gyrase

In this study, all-atom MD simulations were conducted to validate the top-scored docked conformations of the complexes. We evaluated dynamic stability using measures such as the backbone *RMSD*, *Cα-RMSF*, *Rg*, and *SASA* profiles over a 200-ns time scale.

The CIS1, CIS2, CIS5, CIS11, and CIS12–1AD1 complexes had typical backbone RMSD values of 0.27 ± 0.03 nm, 0.23 ± 0.04 nm, 0.25 ± 0.05 nm, 0.32 ± 0.07 nm, and 0.27 ± 0.05 nm, respectively (Figure 2A). The RMSD values for the CIS5 and CIS12-2INR complexes were 0.24 ± 0.05 nm and 0.19 ± 0.04 nm, respectively (Figure 2D), whereas the RMSD values for CIS1, CIS4, and CIS5-3G7B were 0.15 ± 0.04 nm, 0.10 ± 0.01 nm, and 0.12 ± 0.02 nm, respectively (Figure 2G). Most complexes displayed stable RMSD trends during the last 100 ns, with CIS5 exhibiting the most constant behaviour across all the systems (Rout et al., 2023).

**FIGURE 3:**
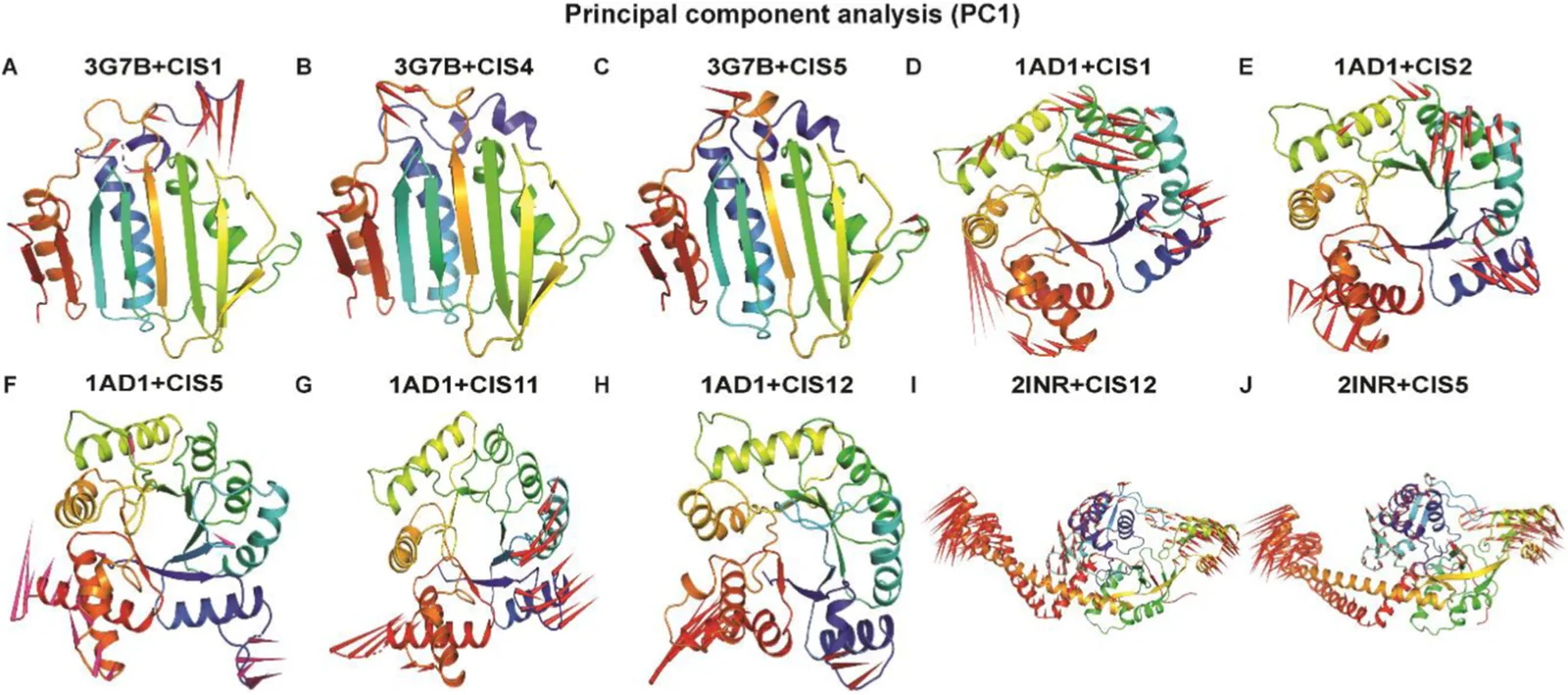
Porcupine plots displaying the motions represented by the top principal component (PC), i.e., PC1, in S. aureus DNA gyrase and dihydropteroate synthase-ciprofloxacin-sulfonamide hybrid derivative complexes. The protein is shown in cartoon representation, while the porcupine arrows indicate the direction and magnitude of the dominant motions associated with PC1. **(A–C)** Porcupine plots showing PC1-associated motions in the dihydropteroate synthase (DHPS, 1AD1)-ciprofloxacin-sulfonamide hybrid derivative complexes. **(D–F)** PC1-associated motions in the DNA gyrase (2INR)-ciprofloxacin-sulfonamide hybrid derivative complexes. **(G–I)** PC1-associated motions in the DNA gyrase (3G7B)-ciprofloxacin-sulfonamide hybrid derivative complexes.

For the 1AD1 complexes, the average Rg values were approximately 1.88 nm, and those of the 2INR complexes and 3G7B complexes were approximately 2.99 nm and 1.66 nm, respectively (Figures 2B,E,H). All the complexes exhibited constant compactness, with minor variations during the first 50 ns, and stability over the final 100 ns. By measuring the average RMSF of Cα atoms, we investigated residue flexibility. For the 1AD1 complexes, the RMSF values were between 0.10 ± 0.07 and 0.12 ± 0.08 nm, between 0.12 ± 0.06 and 0.13 ± 0.06 nm (Figure 2C), and between 0.06 ± 0.03 and 0.07 ± 0.05 nm for the 2INR complexes (Figure 2F) and 3G7B, respectively (Figure 2I). There were noticeable variations at the N-terminus of 3G7B and the C-terminus of 1AD1.

The SASA values varied from approximately 141–143 nm^2^ for the 1AD1-ligand complexes, from approximately 238–239 nm^2^ for the 2INR-ligand complexes, and from approximately 101–102 nm^2^ for the 3G7B-ligand complexes. Throughout the 200 ns simulation, there were no appreciable variations in SASA, suggesting constant solvent exposure(Rout et al., 2024) (Supplementary Figure S2).

In conclusion, trajectory analysis and dynamic data (Supplementary Table S2) revealed stable complexes with retention of the ligand-bound state, which was confirmed by docking analysis.

### 3.3 Principal component analysis of receptor–ligand complex dynamics

The last 100 ns of the MD trajectories were used to perform principal component analysis (PCA), which examined the collective motion of each receptor-ligand system (Panigrahi, 2008). Covariance matrices were constructed using the protein’s main-chain atoms, which utilize eigenvectors (EVs) and eigenvalues to capture global atomic movements. While the eigenvectors characterize the overall direction of motion of the atoms in each system, the eigenvalues represent the contributions of the atoms to motion (Sahoo et al., 2025). Using the first two principal components (PC1 and PC2) obtained from each system’s PCA, a scatter plot was produced. The trace values for the CIS1, CIS2, CIS5, CIS11, and CIS12–1AD1 complex systems were (26.10, 21.60, 16.91, 23.28, 23.26) nm^2^, respectively, those for the CIS5 and CIS12-2INR complex systems were (40.28 and 36.83) nm^2^, and those for the CIS1, CIS4, and CIS5-3G7B complex systems were (6.26, 4.59, 3.99) nm^2^, according to the covariance matrices that corresponded to the main-chain atoms.

Perhaps as a result of the increased mobility of the ligand within the binding site and the significant contributions from the N-terminal region, the activity of the CIS5-3G7B complex was the lowest, followed by that of CS4-3G7B, CIS1-3G7B, and CIS5-1AD1. CIS4-2INR, which had somewhat higher trace values. According to the PCA plot, these results indicate that the CIS5-3G7B, CIS4-3G7B, CIS1-3G7B, and CIS5-1AD1 complexes adopt stable, well-defined conformations in phase space. The 2D projections of the first two eigenvectors into phase space provided further evidence of the compact structure of all the complexes.

The extreme projections of both eigenvectors were used to create porcupine plots to better visualize the direction of motion captured by the top two eigenvectors (Figure 3; Supplementary Figure S3). For the main-chain atoms, the length of the arrow indicates the strength of the movement, whereas its direction indicates the direction of motion. In these complexes, the C-terminal areas of the CIS5 and CIS12-2INR complexes mostly contribute to the large movements observed, whereas the C-terminal domains exhibit minor movements in the CIS1, CIS4, and CIS5-3G7B complexes. Greater N-terminal domain motions are observed in the CIS1, CIS2, CIS5, CIS11, and CIS12–1AD1 complex systems.

These outcomes align with the conclusions of the RMSF analysis. The CIS1, CIS4, CIS5-3G7B and CIS5-1AD1 complexes show the least amount of mobility and volatility at the amino acid level, according to all of the analyses.

### 3.4 Intermolecular interactions and hydrogen bond occupancy in protein–ligand complexes

Protein‒ligand complexes involve hydrogen bonds and hydrophobic interactions (Ferenczy and Kellermayer, 2022), and the biological activity of possible therapeutic candidates can be improved by the addition of more hydrophobic atoms (Kumari et al., 2022). variations between docked complexes and MD-simulated systems can be identified by analyzing intermolecular interactions. These variations may result from ligand reorientation within the protein-binding pocket or from structural rearrangements.

**FIGURE 4:**
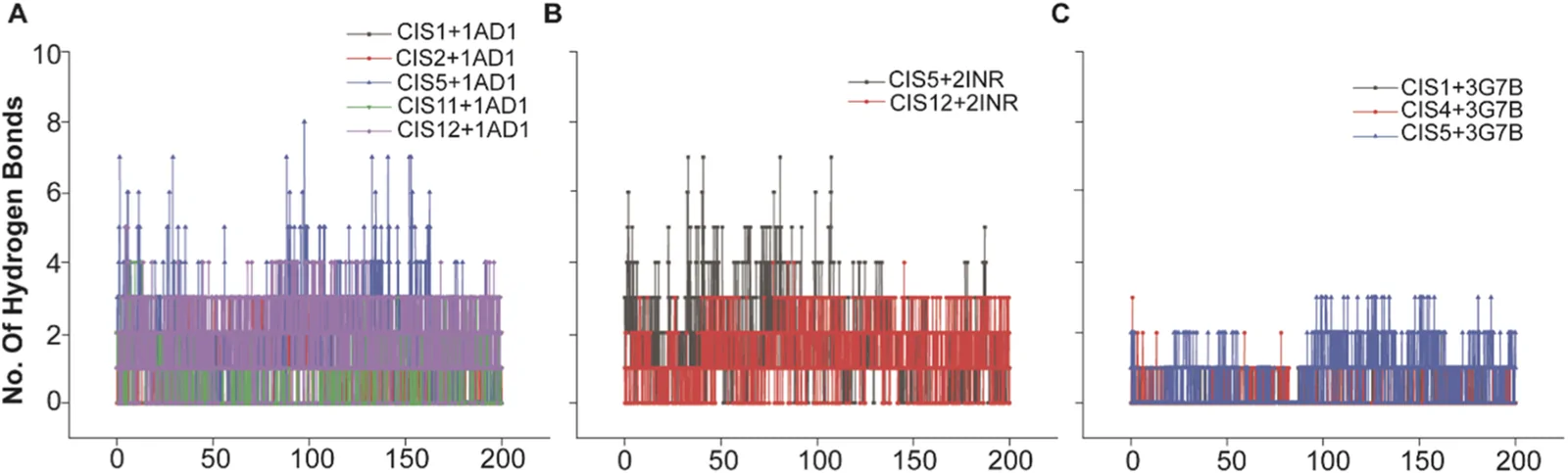
The dynamics of intermolecular hydrogen bonds in complexes formed between S. aureus DNA gyrase and ciprofloxacin derivatives over a time scale of 200 ns. **(A)** DHPS (PDB ID: 1AD1)-ciprofloxacin-sulfonamide hybrid derivative complex. **(B)** DNA gyrase (PDB ID: 2INR)-ciprofloxacin-sulfonamide hybrid derivative complex. **(C)** DNA gyrase (PDB ID: 3G7B)-ciprofloxacin-sulfonamide hybrid derivative complex.

Supplementary Table S2 lists nonbonded interactions, including hydrogen bonds formed during MD, between 1AD1, 2INR, 3G7B, and the screened compounds. The CIS5-2INR and CIS5-3G7B complexes form approximately seven and three hydrogen bonds, respectively, after the CIS5-1AD1 complex, which has a significant binding pocket and forms an average of approximately eight hydrogen bonds per frame (Figure 4. Supplementary Table S2summarizes the donor and acceptor atom hydrogen bond occupancies for 1AD1, 2INR, 3G7B, and the other compounds. The hydrogen bond occupancy of the CIS5-1AD1 complex is greatest at Lys203 (59.59%), Arg52 (79.77%), Arg52 (45.80%), Arg202 (32.47%), and Lys120 (16.58%), followed by Lys39 (34.67%), Lys94 (36.96%), and Arg88 (68.98%) in CIS5-2INR and at Asp81 (17.48%), Glu50 (30.22%), and Glu201 (34.47%) in CIS5-3G7B.

### 3.5 Electro surface potential (ESP) of protein‒ligand complexes

**FIGURE 5:**
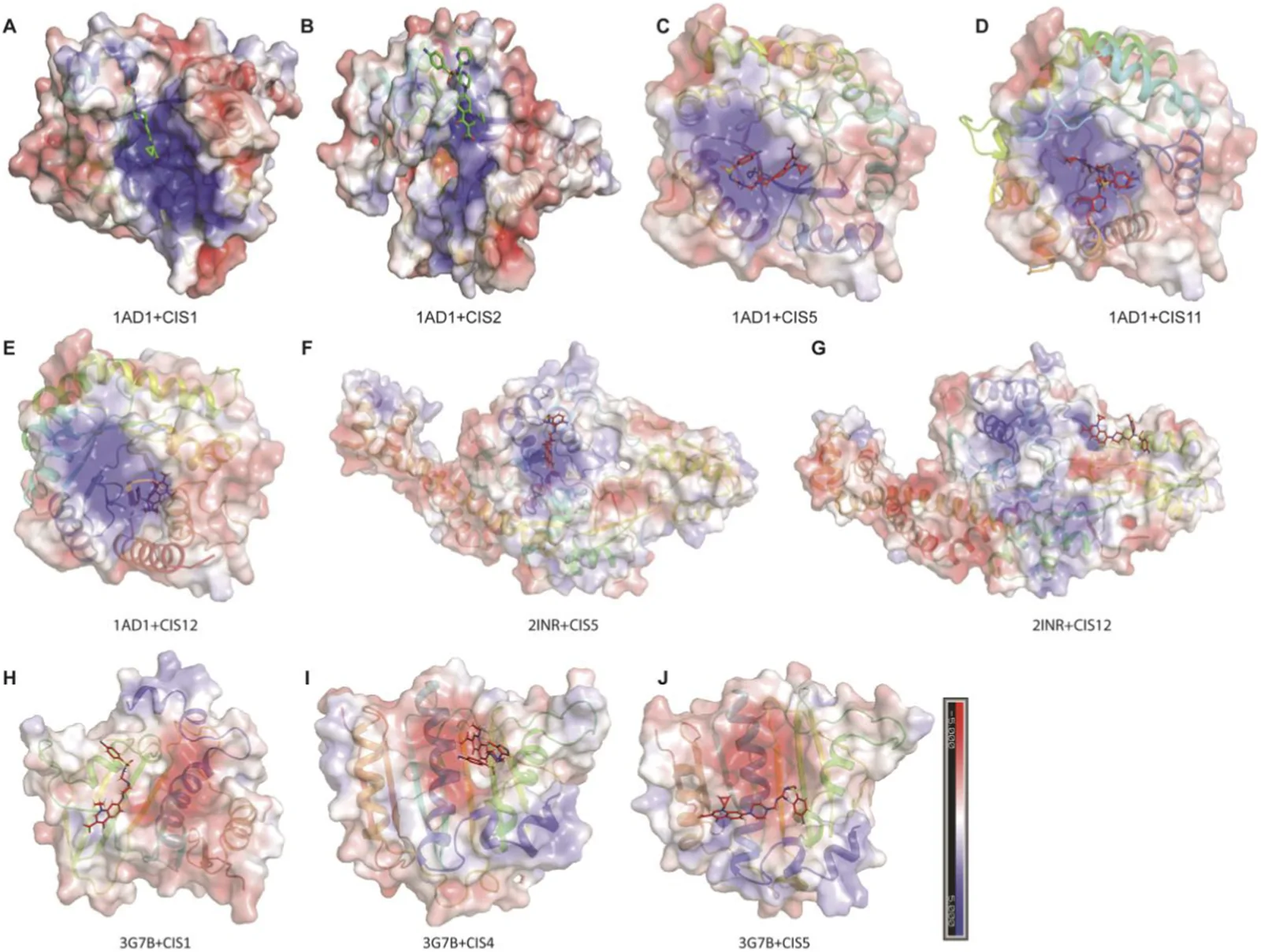
Snapshots of the electrostatic surface potential of the MD-simulated cipro-dihydropteroate synthase and DNA gyrase complexes revealed the observed charge distribution. Snapshots were generated using the APBS plugin in PyMOL. **(A-C)** Electrostatic surface potential (DHPS, PDB: 1AD1). **(D–G)** Electrostatic surface potential (gyrase, PDB:2INR). **(H-J)** Electrostatic surface potential (gyrase, PDB: 3G7B). On this surface, a positive charge represents a blue color, a neutral charge represents a white color, and a negative charge represents a red color.

The key to achieving the best possible affinity and specificity in drug discovery is electrostatic complementarity between the ligand and protein. This method typically involves creating ESP surfaces for multiple ligand sites through visual analysis and comparison, ideally with reference to the ESP structure of the target protein (Rathi et al., 2019; Vascon et al., 2020). Particularly well known is the ability of molecular electrostatics to predict a molecule’s chemical reactivity and its propensity to form specific interactions. ESP surfaces are frequently used to illustrate the electrostatic characteristics of molecules. To create a molecular ESP surface, a unit charge is purposefully transported over its surface. The ESP energy of each surface point is measured, and the surface of the molecule is colored accordingly (Figure 5).

### 3.6 Evolution of 2D-structural elements

Time-dependent changes in secondary structural components within each ciprofloxacin complex were examined using the timeline viewer tool in VMD during a 200 ns molecular dynamics simulation (Panda et al., 2025). Our results demonstrate that ligand interactions modify the binding site of the investigated receptors and induce subtle conformational changes. Time-dependent changes in the secondary structural components of the DHPS and DNA gyrase complexes were evaluated using computational simulations. Interestingly, coils, twists, and loops are more flexible than α-helices and β-sheets, which are hard.

**FIGURE 6:**
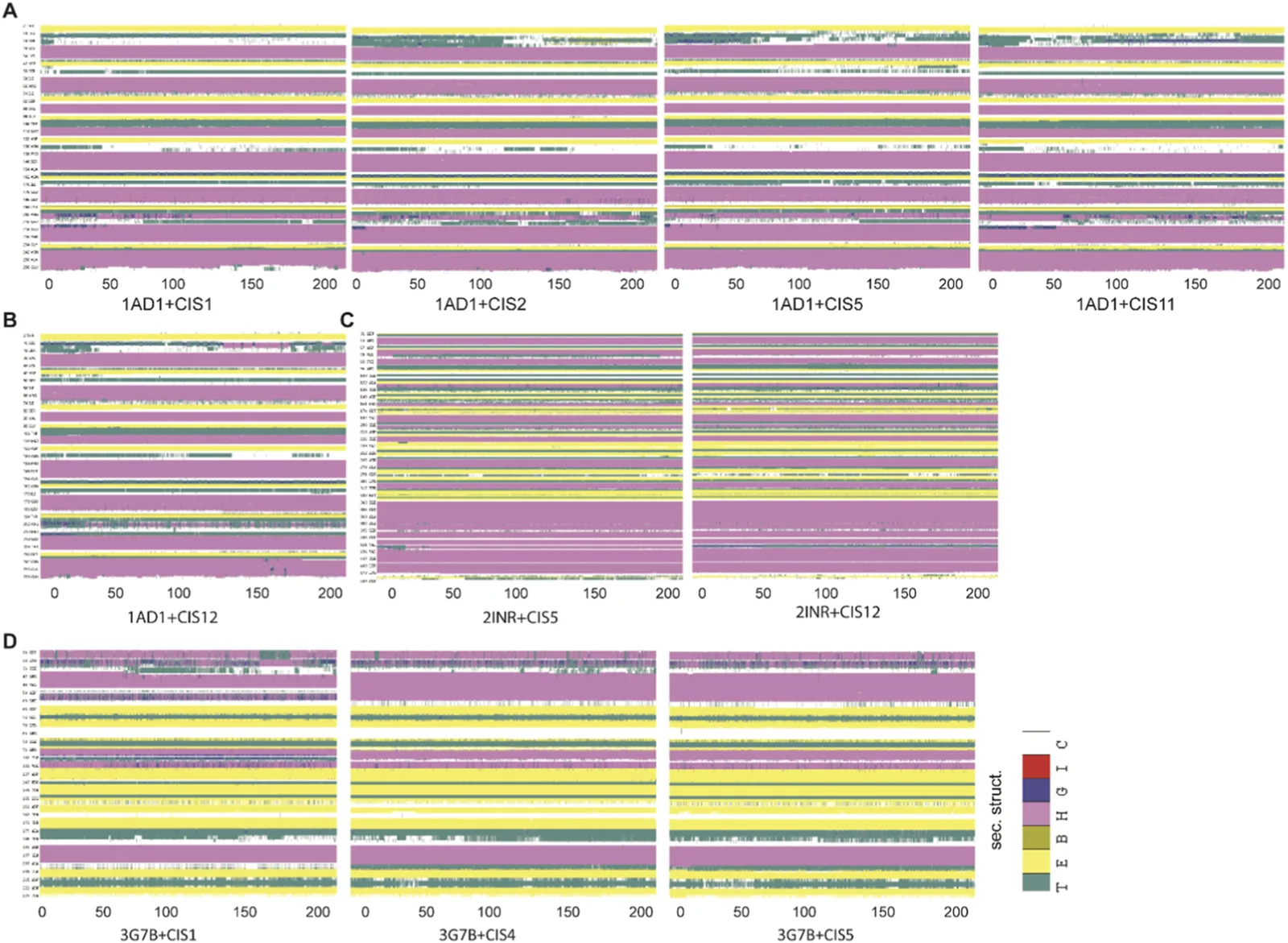
The investigation of changes in the secondary structural elements of bacterial DNA gyrase over a period of 200 ns. The image was generated using the VMD Timeline Viewer, which represents different secondary structural elements, including turns, β-sheets, extended strands, α-helices, π-helices, and coils. **(A)** DHPS (PDB ID: 1AD1)-ciprofloxacin-sulfonamide hybrid derivative complexes CIS1, CIS2, CIS5, and CIS11. **(B)** DHPS (PDB ID: 1AD1)-ciprofloxacin-sulfonamide hybrid derivative complex CIS12. **(C)** DNA gyrase (PDB ID: 2INR)-ciprofloxacin-sulfonamide hybrid derivative complexes CIS5 and CIS12. **(D)** DNA gyrase (PDB ID: 3G7B)-ciprofloxacin-sulfonamide hybrid derivative complexes CIS1, CIS4, and CIS5.

Analysis of structural alterations was performed over a 200-ns duration Figure 6. A thorough analysis of the α-helices revealed that all the 1AD1-CIS complexes had greater occupancy levels than did the 1AD1-CIS12 complexes (Figure 6A). Conversely, a loss of turning from Asn130 to Pro138 in the (1AD1 + CIS12) complex is shown in Figure 6B. More noticeable structural alterations, such as loss of helicity at Ser395 and Glu489 and a loss of turning from Glu291 to Pro161 (Figure 6C), were observed in the DHPS complexes (2INR + CIS5 and 2INR + CIS12). In the 3G7B complexes, larger changes were also observed at Leu60, Arg84, Thr167, and Ala203, as was the development of β-sheets with Asp137, Tyr149, and Thr173 residues (Figure 6D).

The continual development of data collection technologies and modeling approaches creates a recurring difficulty in describing ever-massive molecular data sets in terms of atomic amounts and time intervals. Therefore, improving visualization algorithms remains a potential avenue for future research (Kozlíková et al., 2017). The recent development of drug repositioning is a novel approach for discovering new indications for approved drugs using advanced bioinformatics tools, thereby improving the rational, traditional drug discovery and development to modern drug discovery and development (Zhao et al., 2026; Zhao et al., 2022).

## 4 Conclusion

The increasing prevalence of antibiotic resistance necessitates the identification of novel inhibitors that target essential bacterial enzymes, such as DNA gyrase and dihydropteroate synthase (DHPS). In this study, an integrated computational approach involving molecular docking, all-atom MD simulations, principal component analysis (PCA), and free-energy landscape (FEL) analysis was employed to evaluate the binding behavior of ciprofloxacin-sulfonamide hybrids against these targets. Molecular docking revealed favorable binding affinities of the designed derivatives, with key interactions involving Glu50 and Glu201 in 3G7B, Arg52 and Lys203 in 1AD1, and Arg88 and Lys94 in 2INR. Furthermore, MD simulations demonstrated that the top-performing complexes, namely, CIS5-1AD1, CIS12–1AD1, CIS5-2INR, CIS4-3G7B, and CIS5-3G7B, maintained stable binding conformations throughout the simulation period. PCA further revealed distinct conformational motions within the protein-ligand complexes, consistent with the dynamic behavior observed during the simulations. Overall, the computational findings identify CIS4, CIS5, and CIS12 as promising candidates for further investigation. However, these observations are based solely on *in silico* analyses and require experimental validation through *in vitro* and *in vivo* studies to confirm their antibacterial efficacy and dual-target inhibitory potential. This study provides valuable structural insights and a computational framework for the rational design and prioritization of fluoroquinolone-based antibacterial agents against drug-resistant bacterial pathogens.

## Data Availability

The original contributions presented in the study are included in the article/Supplementary Material, further inquiries can be directed to the corresponding authors.
