# Supplementary material for: All-atoms MD simulations study of newly designed ciprofloxacin derivatives as potential bacterial DHPS and DNA gyrase inhibitors
Source: Front Bioinform. 2026 Sep 3;6:1876643. doi: 10.3389/fbinf.2026.1876643 (PMC13582570; doi:10.3389/fbinf.2026.1876643)
Supplement: Supplementary file 1 [file DataSheet1.pdf]

Supporting Information

All-atoms MD simulations study of newly designed ciprofloxacin derivatives as potential bacterial DHPS and DNA gyrase inhibitors

Chita Ranjan Sahoo[a], Madhusmita Rout[a], Sudhir Kumar Paidesetty[b], Budheswar Dehury[c]*, Debdutta Bhattacharya[a]*, Sanghamitra Pati[a,d]

[a]*ICMR-Regional Medical Research Centre, Department of Health Research, Ministry of Health and Family Welfare, Government of India, Bhubaneswar-751023, India*
[b]*Medicinal Chemistry Research Laboratory, School of Pharmaceutical Sciences, Siksha 'O' Anusandhan Deemed to be University, Bhubaneswar, India*
[c]*Department of Bioinformatics, Manipal School of Life Sciences, Manipal Academy of Higher Education, Karnataka, India*
[d]*Indian Council of Medical Research, New Delhi, 110029, India*

***Corresponding Authors**
Dr. B Dehury (budheswar.dehury@manipal.edu)
Dr. D Bhattacharya (drdebdutta.bhattacharya@yahoo.co.in)

Sl No	Content	Page No
Table S1.	*Newly designed Ciprofloxacin derivatives and their detailed 2D confirmers*	***Page S3-4***
Table S2	*Stability of Ciprofloxacin derivatives with bacterial protein (PDBID: 1AD1), (PDBID: 2INR) and (PDBID: 3G7B) complexes computed using during timescale 200 ns trajectories of each system*	***Page S5***
Table S3	*The hydrogen-bond occupancy of the residues involved in the interaction inside ciprofloxacin derivative complexes assessed for 200ns molecular dynamics simulation*	***Page S6***
Figure S1	*The non-bonded 2D interaction between ciprofloxacin derivatives with DHPS and DNA gyrase by analyzing the top-ranked complexes obtained from molecular docking studies*	***Page S7***
Figure S2	*The investigation of solvent-accessible area ligand-protein interaction at 200 nanoseconds.*	***Page S8***
Figure S3	*Principal component analysis projecting motion occupied of the top-ranked PC2 and the porcupine plot projecting with inward-outward S. aureus and dihydropteroate synthase-cipro derivative complex.*	***Page S9***

Table S1. Newly designed Ciprofloxacin derivatives and their detailed 2D confirmers

Sl No.	Compounds	Structure
1	CIS1 (Ciprofloxacin + Sulfanilamide)	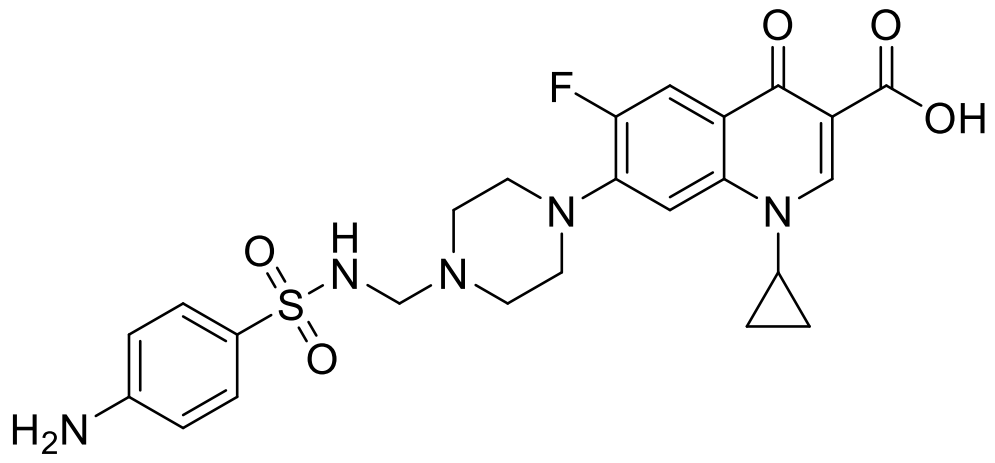
2	CIS2 (Ciprofloxacin + Sulfadiazine)	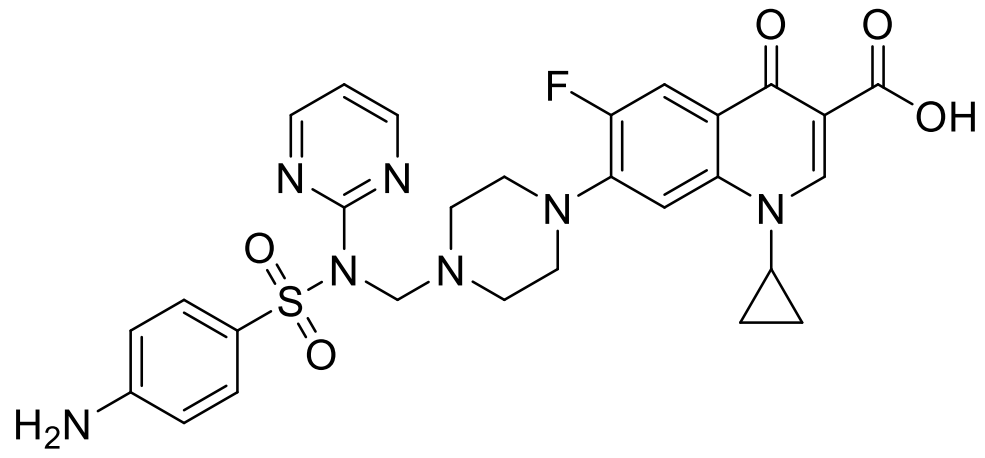
3	CIS3 (Ciprofloxacin + Sulfapyridine)	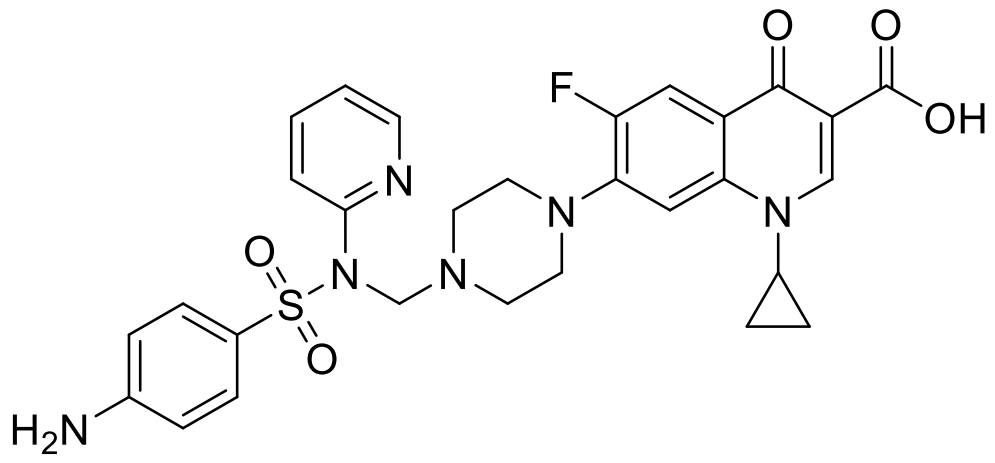
4	CIS4 (Ciprofloxacin + Sulfamethoxazole)	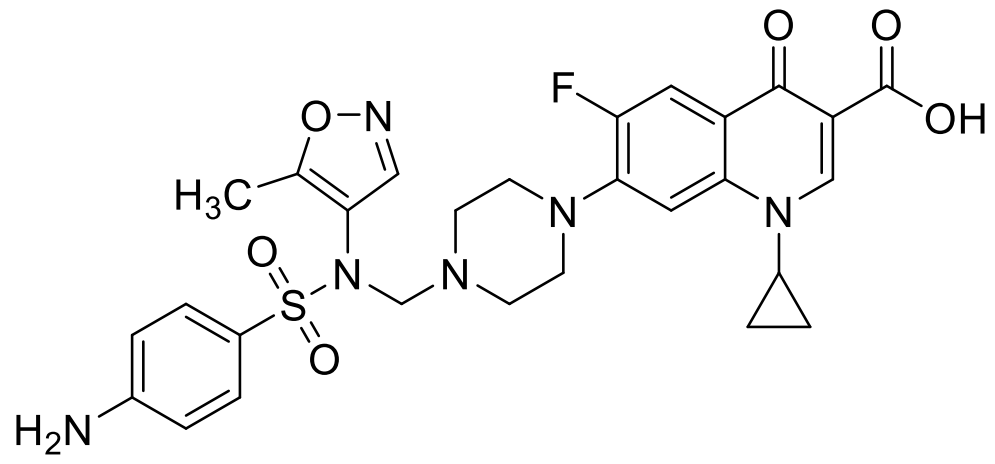
5	CIS5 (Ciprofloxacin + Sulfaguanidine)	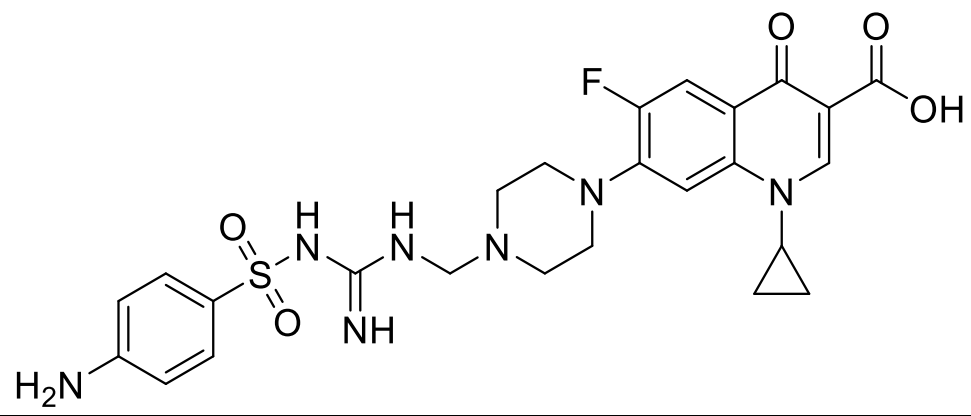
6	CIS6 (Ciprofloxacin + Sulfacetamide)	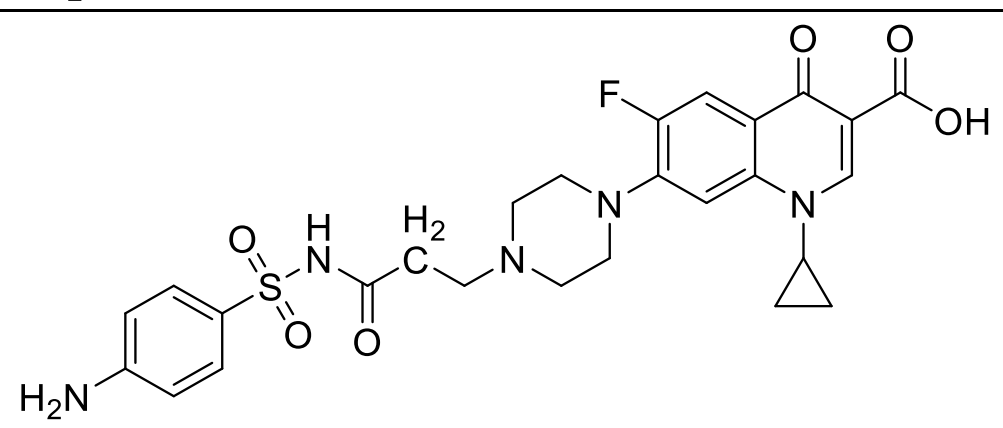
7	CIS7 (Ciprofloxacin + Sulfathiazole)	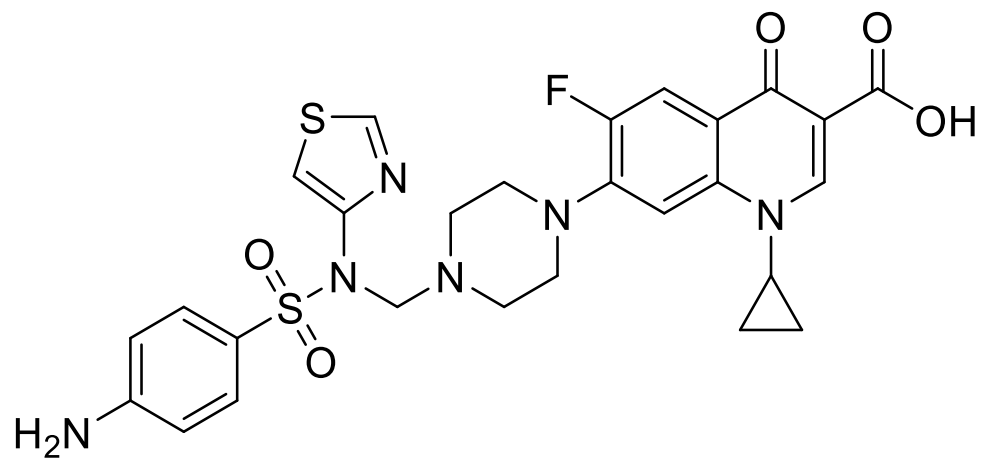

8	CIS8 (Ciprofloxacin + Sulfaoxazole)	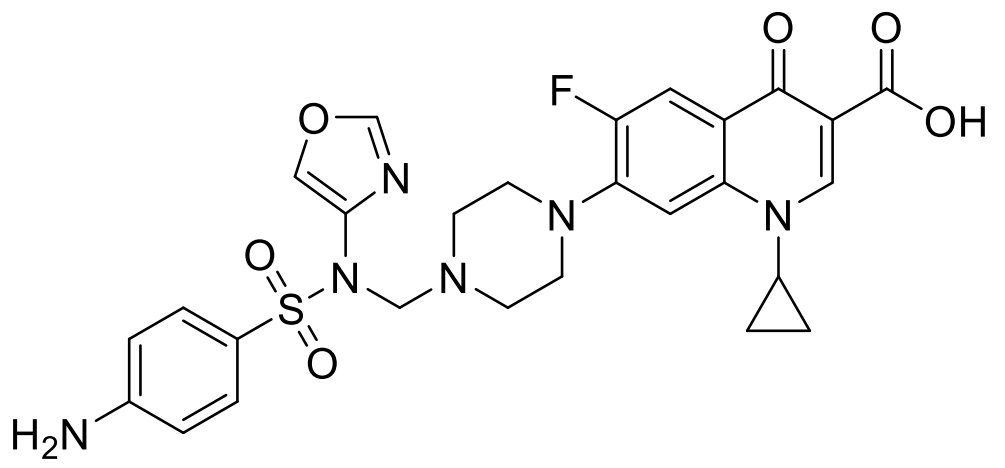
9	CIS9 (Ciprofloxacin + Sulfametiazole)	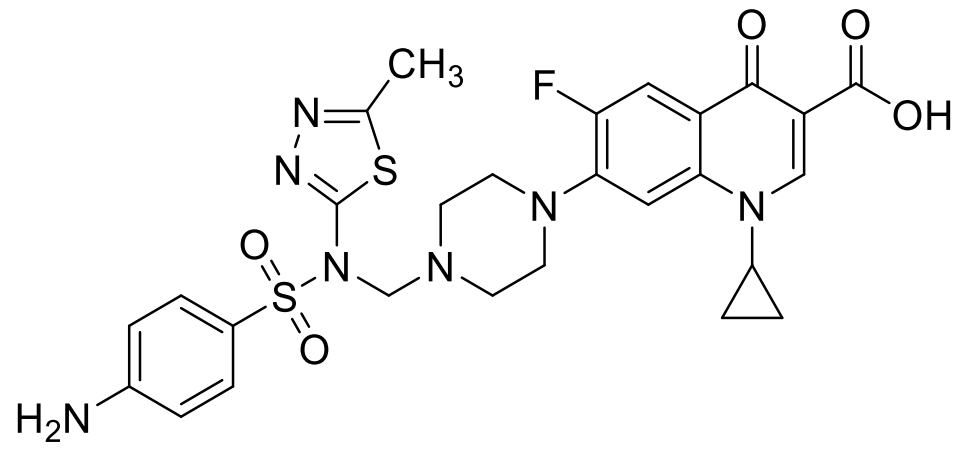
10	CIS10 (Ciprofloxacin + Sulfadimidine)	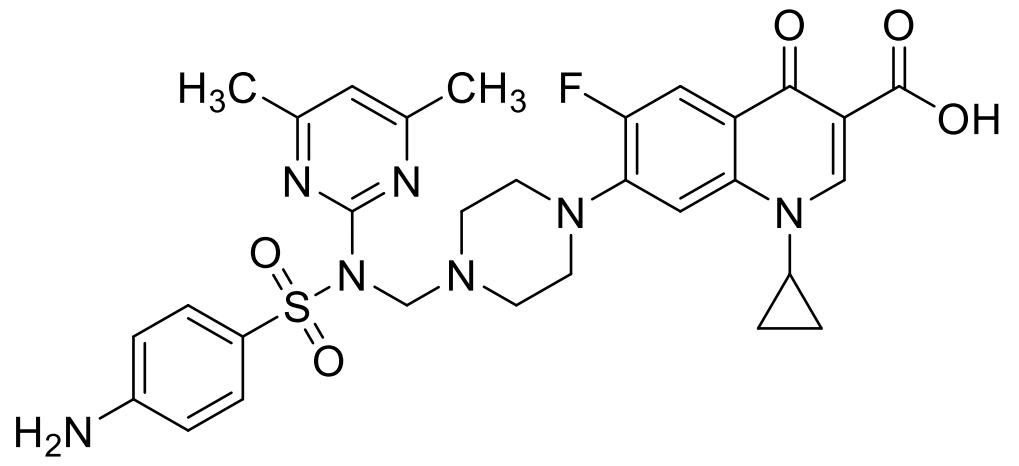
11	CIS11 (Ciprofloxacin + Sulfadoxine)	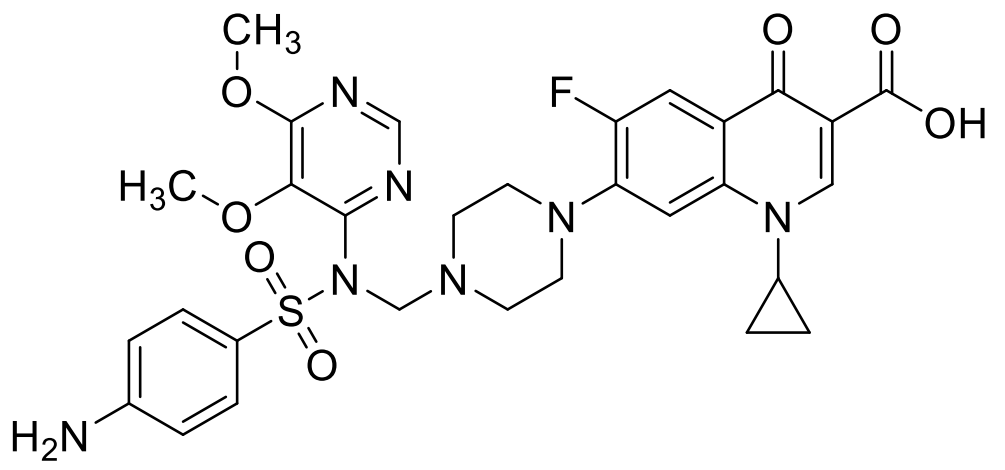
12	CIS12 (Ciprofloxacin + Sulfamerazine)	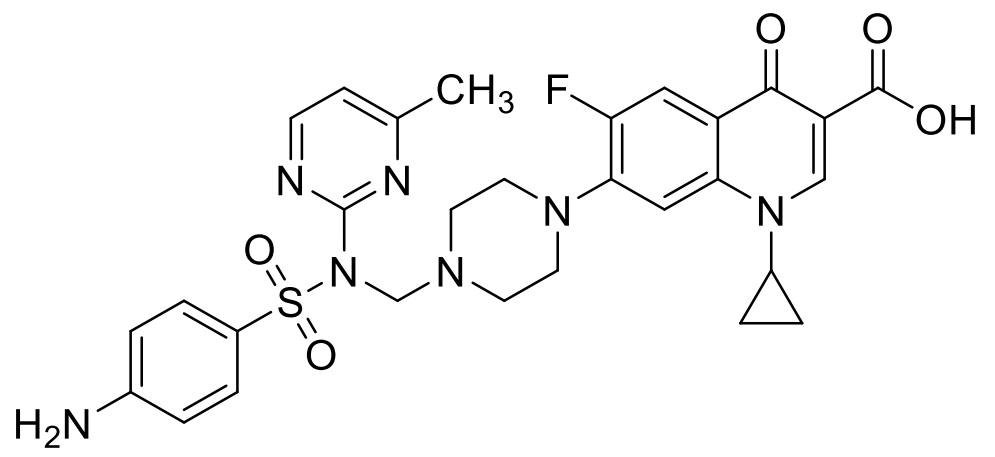
13	CIS13 (Ciprofloxacin + Sulfafurazole)	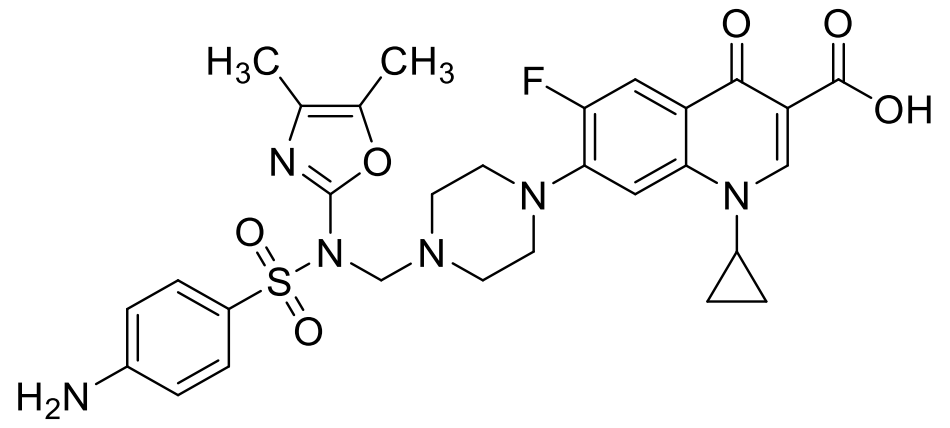
14	CIS14 (Ciprofloxacin + Sulfamethoxydiazine)	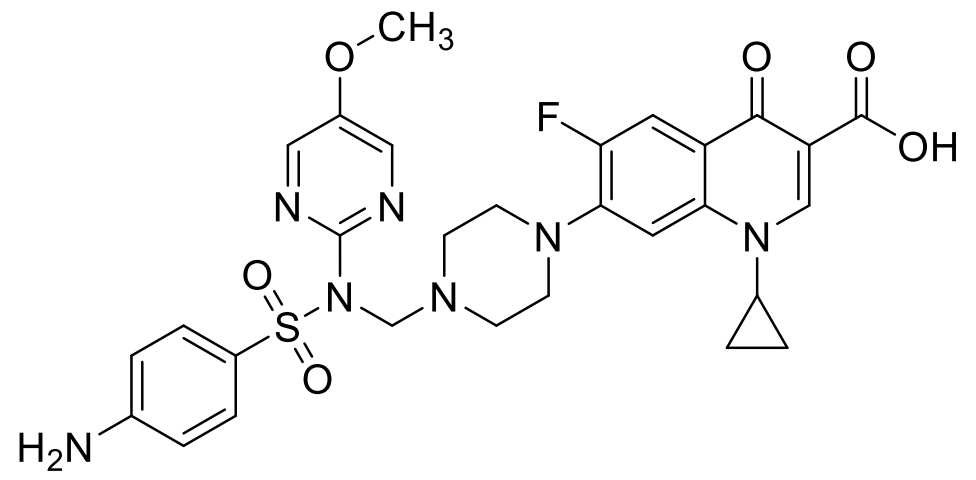
15	CIS15 (Ciprofloxacin + Sulfalene)	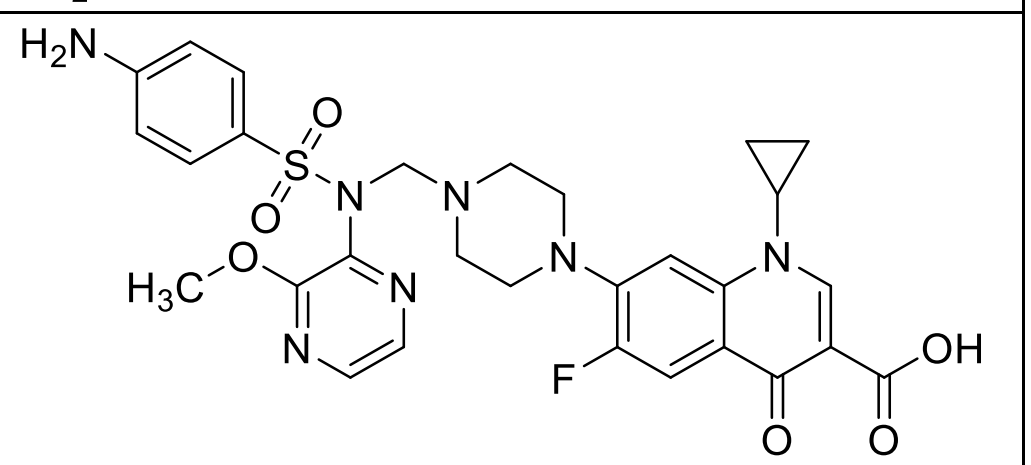

Table S2. Stability of Ciprofloxacin derivatives with bacterial protein (PDBID: 1AD1), (PDBID: 2INR) and (PDBID: 3G7B) complexes computed using during timescale 200 ns trajectories of each system

Compound	*1AD1*			
	RMSD (nm)	*Rg (nm)*	*RMSF (nm)*	*SASA (nm^2)*
CIS1	*0.27±0.03*	*1.90±0.02*	*0.11±0.10*	*143.20±3.86*
CIS2	*0.23±0.04*	*1.90±0.02*	*0.11±0.08*	*143.92±5.36*
CIS5	*0.25±0.05*	*1.88±0.02*	*0.10±0.07*	*140.07±3.22*
CIS11	*0.32±0.07*	*1.88±0.02*	*0.12±0.08*	*143.25±3.85*
CIS12	*0.27±0.05*	*1.88±0.01*	*0.11±0.09*	*141.14±3.09*
Compound	*2INR*			
	RMSD (nm)	*Rg (nm)*	*RMSF (nm)*	*SASA (nm^2)*
CIS5	*0.24±0.05*	*2.99±0.02*	*0.13±0.06*	*239.39±2.49*
CIS12	*0.19±0.04*	*3.01±0.02*	*0.12±0.06*	*238±2.58*
Compound	*3G7B*			
	RMSD (nm)	*Rg (nm)*	*RMSF (nm)*	*SASA (nm^2)*
CIS1	*0.15±0.04*	*1.66±0.007*	*0.07±0.05*	*102.47±1.67*
CIS4	*0.10±0.01*	*1.67±0.006*	*0.07±0.03*	*102.49±1.52*
CIS5	*0.12±0.02*	*1.66±0.009*	*0.06±0.033*	*101.49±1.57*

Table S2. The hydrogen-bond occupancy of the residues involved in the interaction inside ciprofloxacin derivative complexes assessed for 200ns molecular dynamics simulation

Receptor	Complex	Residues exhibiting hydrogen bond occupancy (%)
**1AD1**	*CIS1+1AD1*	*Arg202(2.15), Lys207(7.84), Arg219 (9.49), Lys233(10.19),* ***Lys120(16.58)***
	CIS2+1AD1	*Arg219(3.15), Ser18(4.55), Lys207(18.68),* ***Arg202(32.47)***
	CIS5+1AD1	*Arg202(7.79), Lys203(45.25),* ***Arg52(45.80)***
	CIS11+1AD1	*Arg239(5.00), Glu27(5.39)*
	CIS12+1AD1	*Glu218(9.34),* ***Lys203(59.59), Arg52(79.77),***
**2INR**	*CIS5+2INR*	*Glu246(2.00), Ile (2.10), Arg249(4.90), Arg88(6.64), Ser172(16.28), Ala30(21.58),* ***Lys39(34.67)***
	CIS12+2INR	*Ser172(3.15), Lys39(4.40),* ***Lys94(36.96) Arg88(68.98)***
**3G7B**	*CIS1+3G7B*	*Arg84(2.00)*
	CIS4+3G7B	*Gln91(6.69),* ***Asp81(17.48)***
	CIS5+3G7B	*Gly24(2.35), Tyr35(2.55),* ***Glu50(30.22), Glu201(34.47)***

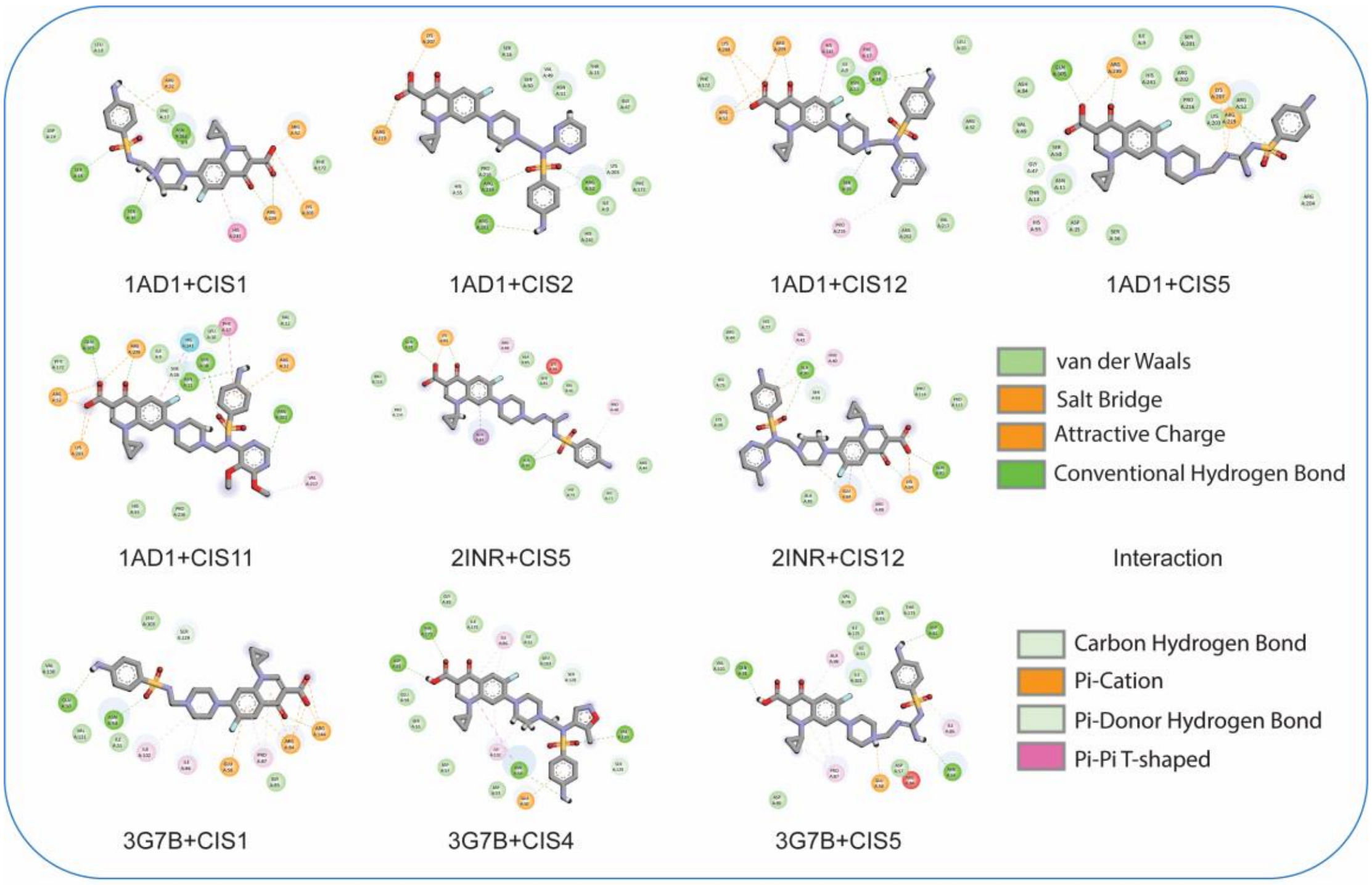

Figure S1. The non-bonded 2D interaction between ciprofloxacin derivatives with DHPS and DNA gyrase by analyzing the top-ranked complexes obtained from molecular docking studies

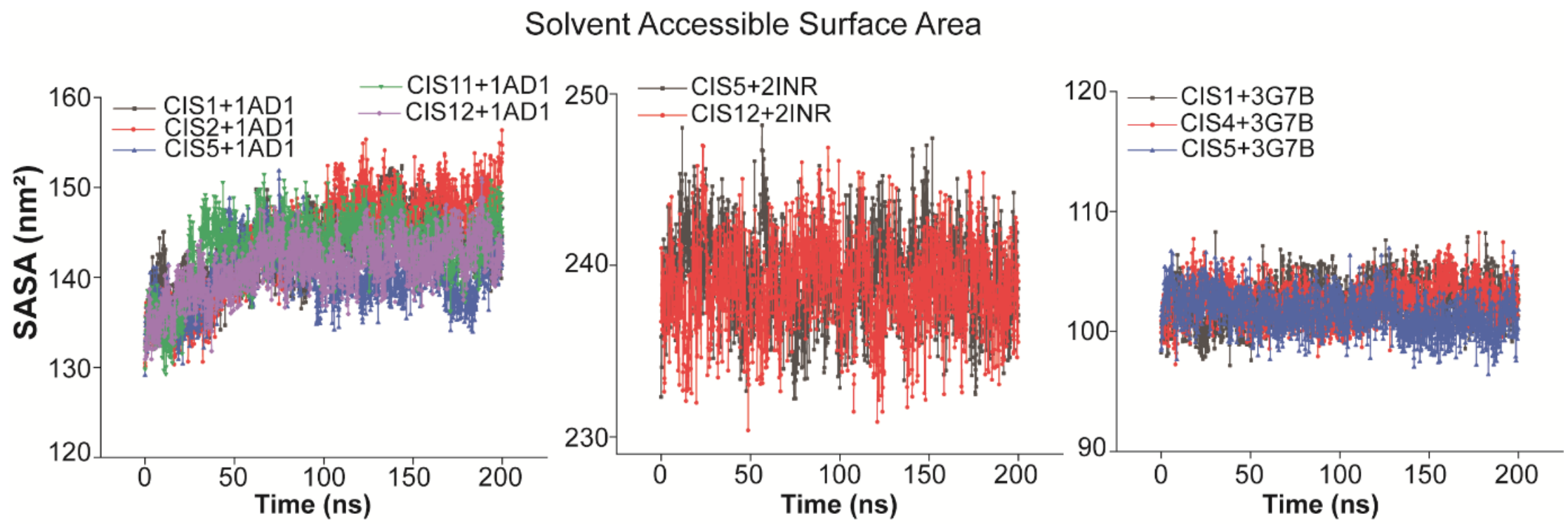

Figure S2. The investigation of solvent-accessible area ligand-protein interaction at 200 nanoseconds.

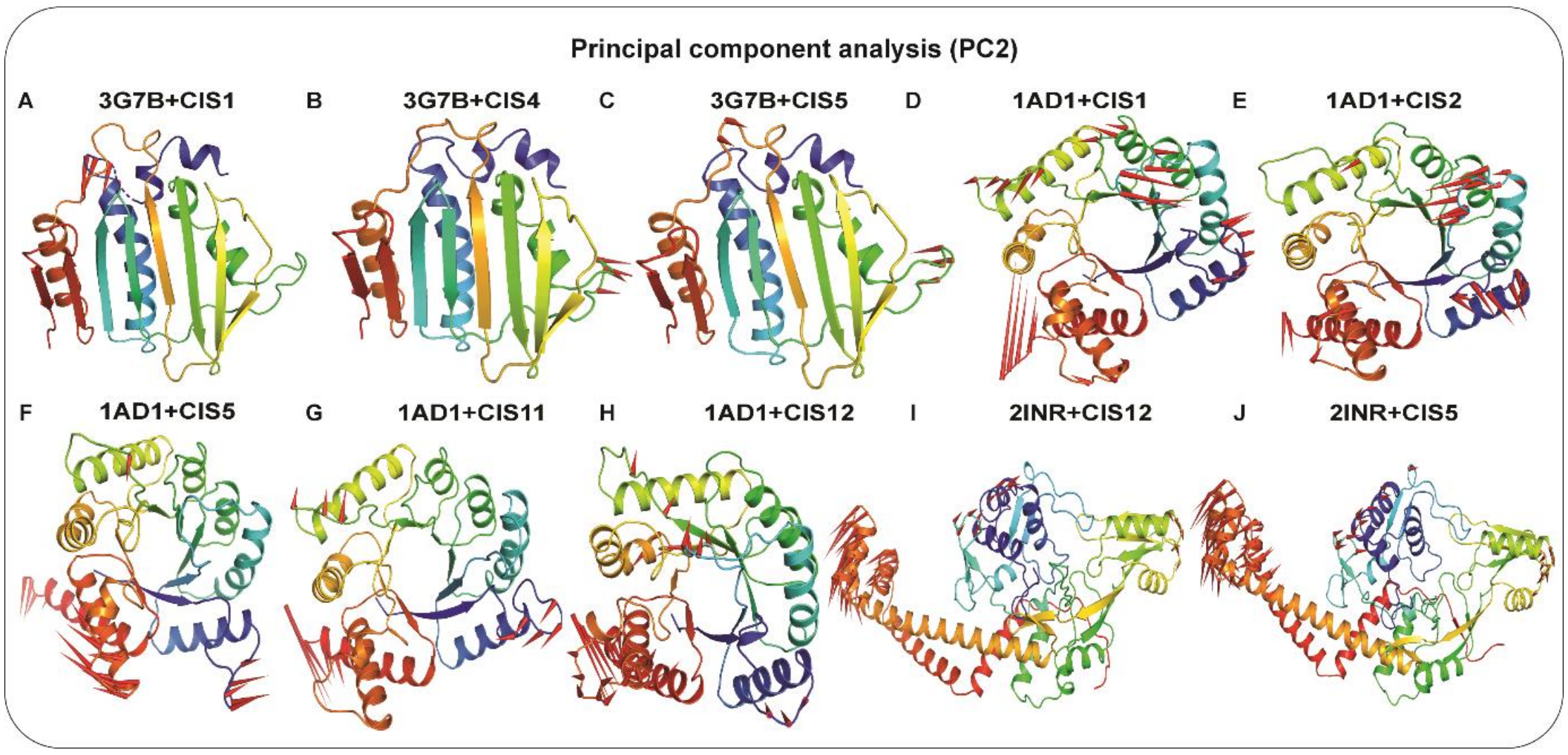

Figure S3. Principal component analysis projecting motion occupied of the top-ranked PC2 and the porcupine plot projecting with inward-outward *S. aureus* and dihydropteroate synthase-cipro derivative complex.
